# Embodied bidirectional simulation of a spiking cortico-basal ganglia-cerebellar-thalamic brain model and a mouse musculoskeletal body model distributed across computers including the supercomputer Fugaku

**DOI:** 10.3389/fnbot.2023.1269848

**Published:** 2023-10-05

**Authors:** Yusuke Kuniyoshi, Rin Kuriyama, Shu Omura, Carlos Enrique Gutierrez, Zhe Sun, Benedikt Feldotto, Ugo Albanese, Alois C. Knoll, Taiki Yamada, Tomoya Hirayama, Fabrice O. Morin, Jun Igarashi, Kenji Doya, Tadashi Yamazaki

**Affiliations:** ^1^Graduate School of Informatics and Engineering, The University of Electro-Communications, Tokyo, Japan; ^2^Neural Computation Unit, Okinawa Institute of Science and Technology Graduate University, Okinawa, Japan; ^3^Image Processing Research Team, Center for Advanced Photonics, RIKEN, Saitama, Japan; ^4^Computational Engineering Applications Unit, Head Office for Information Systems and Cybersecurity, RIKEN, Saitama, Japan; ^5^Robotics, Artificial Intelligence and Real-Time Systems, Faculty of Informatics, Technical University of Munich, Munich, Germany; ^6^Department of Excellence in Robotics and AI, The BioRobotics Institute, Scuola Superiore Sant'Anna, Pontedera, Italy; ^7^Graduate School of Information Science and Technology, The University of Tokyo, Tokyo, Japan; ^8^Center for Computational Science, RIKEN, Hyogo, Japan

**Keywords:** spiking neural networks, musculoskeletal model, distributed simulation, Fugaku, Neurorobotics Platform, ROS

## Abstract

Embodied simulation with a digital brain model and a realistic musculoskeletal body model provides a means to understand animal behavior and behavioral change. Such simulation can be too large and complex to conduct on a single computer, and so distributed simulation across multiple computers over the Internet is necessary. In this study, we report our joint effort on developing a spiking brain model and a mouse body model, connecting over the Internet, and conducting bidirectional simulation while synchronizing them. Specifically, the brain model consisted of multiple regions including secondary motor cortex, primary motor and somatosensory cortices, basal ganglia, cerebellum and thalamus, whereas the mouse body model, provided by the Neurorobotics Platform of the Human Brain Project, had a movable forelimb with three joints and six antagonistic muscles to act in a virtual environment. Those were simulated in a distributed manner across multiple computers including the supercomputer Fugaku, which is the flagship supercomputer in Japan, while communicating via Robot Operating System (ROS). To incorporate models written in C/C++ in the distributed simulation, we developed a C++ version of the rosbridge library from scratch, which has been released under an open source license. These results provide necessary tools for distributed embodied simulation, and demonstrate its possibility and usefulness toward understanding animal behavior and behavioral change.

## 1. Introduction

To investigate the neural mechanisms underlying behavioral changes, it is crucial to examine how neural activity is altered by learning through interaction with the environment via a physical body. Bidirectional computer simulations incorporating both brain and body models offer a promising approach for reproducing and predicting neural activity changes during learning and subsequent behavioral modifications. However, such brain-body-environment simulations can be exceedingly complex and large, necessitating a distributed simulation system in which multiple computers participate over the Internet. Several technologies have been developed to realize such distributed simulations (Quigley et al., 2009; Djurfeldt et al., 2010), and a distributed brain-body-environment simulation has been already demonstrated (Feldotto et al., 2022a) using the supercomputer Piz Daint within a unified framework for embodied simulation called the Neurorobotics Platform (NRP) developed under the Human Brain Project (Knoll and Gewaltig, 2016).

To extend the size and details of the model further, we decided to recruit multiple supercomputers with different architectures, separated physically and geographically. We have been using the supercomputer Fugaku, which is the Japanese flagship supercomputer (RIKEN, 2021) under the MEXT Program, and have created a large-scale spiking network model of the cortico-basal ganglia-thalamic (CBT) circuit (Gutierrez et al., 2020). Also, we have developed a spiking cerebellar model (CB model) on a Graphics Processing Unit (GPU) cluster (Kuriyama et al., 2021). The NRP officially supports only the Ubuntu operating system, and so a Linux machine specifically running Ubuntu is necessary to use the body-environment platform. While the brain-body-environment system in a previous study (Feldotto et al., 2022a) was completed within a single computer system, we posited that connecting the above three models and running them harmoniously across physically separated system, would enable more flexible simulation. Furthermore, we aimed to provide a tool to realize such distributed simulation.

Robot Operating System (ROS) is a de-facto standard message-passing middleware suite for communicating among robots and controllers called “ROS nodes” in a local network used in the field of robotics (Quigley et al., 2009). On the other hand, to realize distributed simulations beyond a local network, all ROS nodes must be able to communicate with each other across separate networks, which cannot be made by the plain ROS. To address this issue, the rosbridge protocol (ROS, 2022) provides a means to communicate beyond a local network, in which messages are wrapped in JSON format, and are exchanged via WebSocket. Because WebSocket is implemented on https, and https seems to be opened by firewalls at most institutions, the distributed simulation can be realized by rosbridge. Unfortunately, the rosbridge library had only been implemented in Python and Javascript. No C/C++ version was available. Because the CB model has been developed in C/C++ (Kuriyama et al., 2021) to use GPUs, we need a C/C++ version of the rosbridge library.

In the present study, we first developed a C/C++ version of the rosbridge library, and released it under an open source license (Omura, 2022). While the library can be used in various situations in which a rosbridge library is required, in this study, we used the library to conduct embodied bidirectional simulation including a secondary motor cortex (M2) model written in C++ and a CBT model implemented in the NEST simulator on Fugaku, a CB model written in C++ and CUDA (Kuriyama et al., 2021) on a local GPU cluster, and a body-environment model on another local computer. Using this system, we simulated a behavioral task in which a mouse pushes and pulls a lever alternatively, and furthermore, we demonstrated that the mouse gradually adapted an amplitude of the lever movement online by the CB model. Thus, a brain model that consists of different brain regions, and a body and environment model across three computers including Fugaku were coordinated to achieve a bidirectional brain-body-environment simulation.

## 2. Materials and methods

### 2.1. Overview of the system architecture

**Figure 1:**
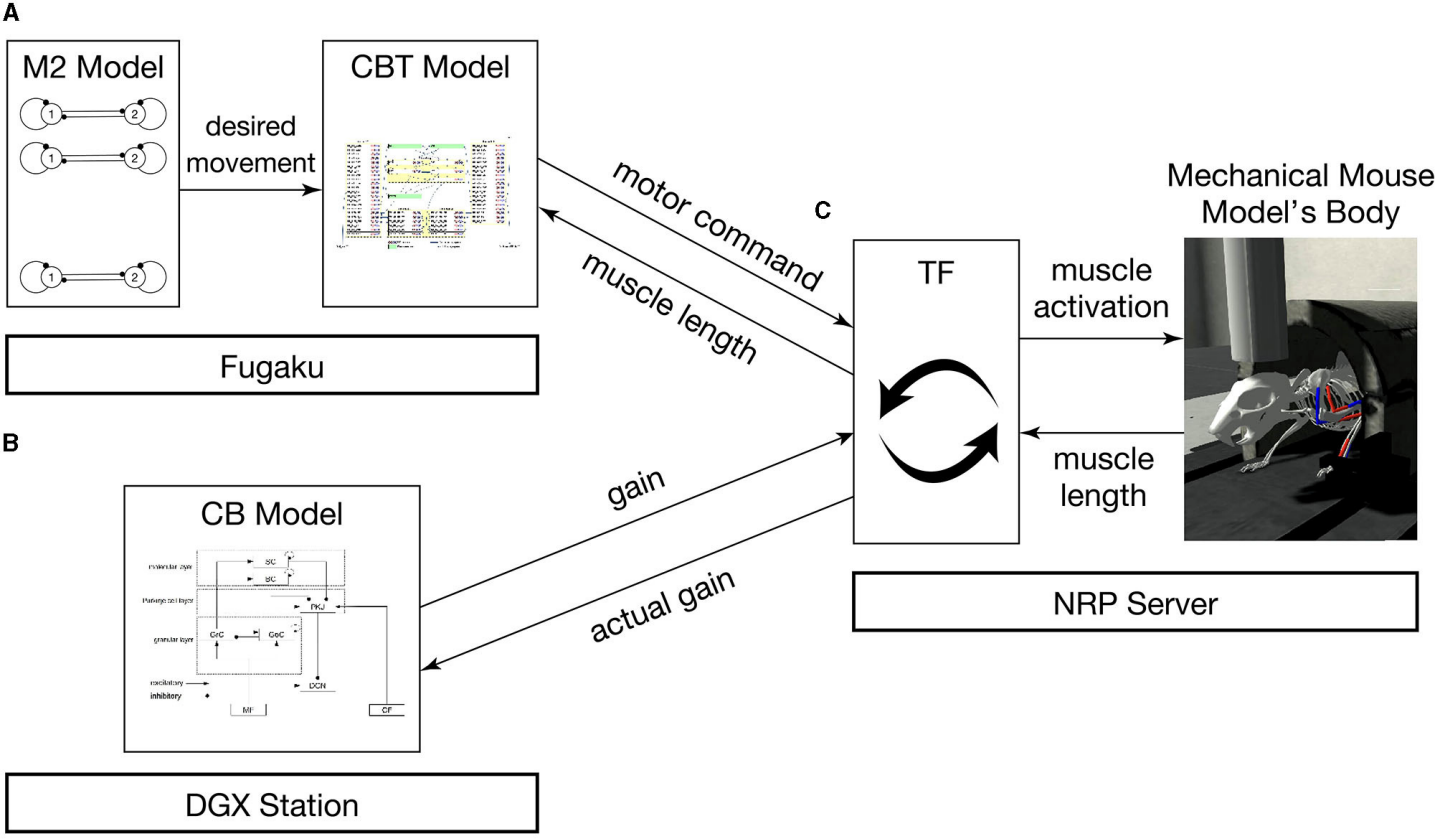
Overview of the simulation models setup. The system consists of **(A)** the M2 model written in C++ and the CBT model implemented in the NEST simulator on Fugaku, **(B)** the GPU-based CB model written in C++ with CUDA running on a local GPU cluster, and **(C)** a mouse musculoskeletal model implemented in NRP on another local computer. In **(A)**, the M2 model sends spike trains to the CBT model. Between **(A)** and **(C)**, the population-averaged firing rate of neurons in the CBT model (L5B PT neurons in M1; Figure 2) is sent to the body, whereas muscle length information is sent back to other neurons (L4 Pyr neurons in S1; Figure 2). Between **(B)** and **(C)**, the compensatory and actual gain information were exchanged. In **(C)**, the body model communicates with the brain model via TF, which implements a fictitious spinal cord model. This TF transforms the firing rate of M1 neurons into muscle activation signals, and computes the actual gain.

The brain-body model consists of a brain model which includes multiple regions and a mouse musculoskeletal body model. The multiple regions in the brain model include an M2 model, a CBT model composed of primary motor (M1) and somatosensory (S1) cortices, basal ganglia (BG) and thalamus (TH), and a CB model. Those models were distributed across multiple computers synchronously while communicating bidirectionally (Figure 1). Specifically, the M2 and CBT models ran on Fugaku, the CB model on a local GPU cluster, and the body-environment model on another local computer called an NRP server. Those models across the three computers communicated with each other while passing messages. The body-environment model was simulated in the Gazebo simulator that uses Simbody as the physics engine. The Gazebo simulator also provided a visualization function. A proxy function called a transfer function (TF), which implemented a fictitious spinal cord model, exchanged messages among the multiple regions in the brain model and the body-environment simulator. The details of the communications are described in Section 2.4.

### 2.2. Brain region models

#### 2.2.1. M2 model

The M2 model was assumed to provide information on desired goal movements to the downstream brain and body. Specifically, the M2 model was built to provide two spike trains that alternate in time to represent periodic movements of the forelimb of the mouse body. Eventually, we developed the M2 model as a set of 100 central pattern generators (CPGs) to generate these alternating spike patterns, where each CPG model was implemented as a Matsuoka oscillator (Matsuoka, 1985) with leaky integrate-and-fire (LIF) neurons as follows:


(1) $$\begin{array}{l} \tau_{\text{m}}\frac{dV_{i}}{dt}\left(t\right)=-\left(V_{i}\left(t\right)-V_{\text{rest}}\right)\\ \;+R_{\text{syn}}I_{\text{syn}i}\left(t\right)-R_{\text{ahp}}I_{\text{ahp}i}\left(t\right)+R_{\text{m}}I_{\text{ext}i}\left(t\right), \end{array}$$

(2) $$\begin{array}{lll} V_{i}\left(t\right)>\theta & \Rightarrow & S_{i}\left(t\right)=1,V_{i}\left(t\right)\leftarrow V_{\text{reset}} \end{array}$$

where *V*_*i*_(*t*) is the membrane potential of neuron *i*, τ_m_ = 20 ms is the membrane time constant, *R*_syn_ = 1.0 MΩ is the resistance of the synapse, *I*_syn*i*_(*t*) is the inhibitory synaptic current, *R*_ahp_ = 0.5 MΩ is the resistance of the adaptation, *I*_ahp*i*_(*t*) is the adaptation current, *R*_m_ = 1.0 MΩ is the resistance of the membrane, *I*_ext*i*_(*t*) is the external input current, θ = −55 mV is the threshold for spike emission, *S*_*i*_(*t*) is the spike event of neuron *i* at time *t*, and *V*_reset_ = −65 mV is the reset potential. When *V*_*i*_(*t*) exceeds θ, a spike is emitted. And then, *V*_*i*_(*t*) is reset to *V*_reset_.

To avoid aligning the firing times at all CPGs, *I*_ext_(*t*) is randomly drawn from a uniform distribution in the range of [−19.95, 20.05] at every time step as background noise. *I*_syn_(*t*), *I*_ahp_(*t*) are described as current-based exponentially-decaying synapses:


(3) $$\begin{array}{l} I_{\text{syn}i}\left(t\right)=w\underset{f\in S_{j}\left(t\right)}{\sum }\exp\left(-\frac{t-t^{\left(f\right)}}{\tau_{\text{syn}}}\right)\text{Θ}\left(t-t^{\left(f\right)}\right), \end{array}$$

(4) $$\begin{array}{l} I_{\text{ahp}i}\left(t\right)=\underset{g\in S_{i}\left(t\right)}{\sum }\exp\left(-\frac{t-t^{\left(g\right)}}{\tau_{\text{ahp}}}\right)\text{Θ}\left(t-t^{\left(g\right)}\right), \end{array}$$

where τ_syn_ = 5.0 ms is the synaptic time constant, τ_ahp_ = 350.0 ms is the adaptation time constant, *w* = −80 is synaptic strength, *S*_*j*_(*t*) is a set of spikes from pre-synaptic neuron *j*, *S*_*i*_(*t*) is a set of self-initiated spikes, Θ(*t*) is the Heaviside step function, and *t*^(*f*)^ is the time of spike *f*. The synaptic delay is set as 0.1 ms. The differential equation is numerically solved using a forward Euler method with a time step of Δ*t* = 0.1 ms. The model was implemented in C++ and the simulation was conducted on a single node of Fugaku.

#### 2.2.2. CBT model

**Figure 2:**
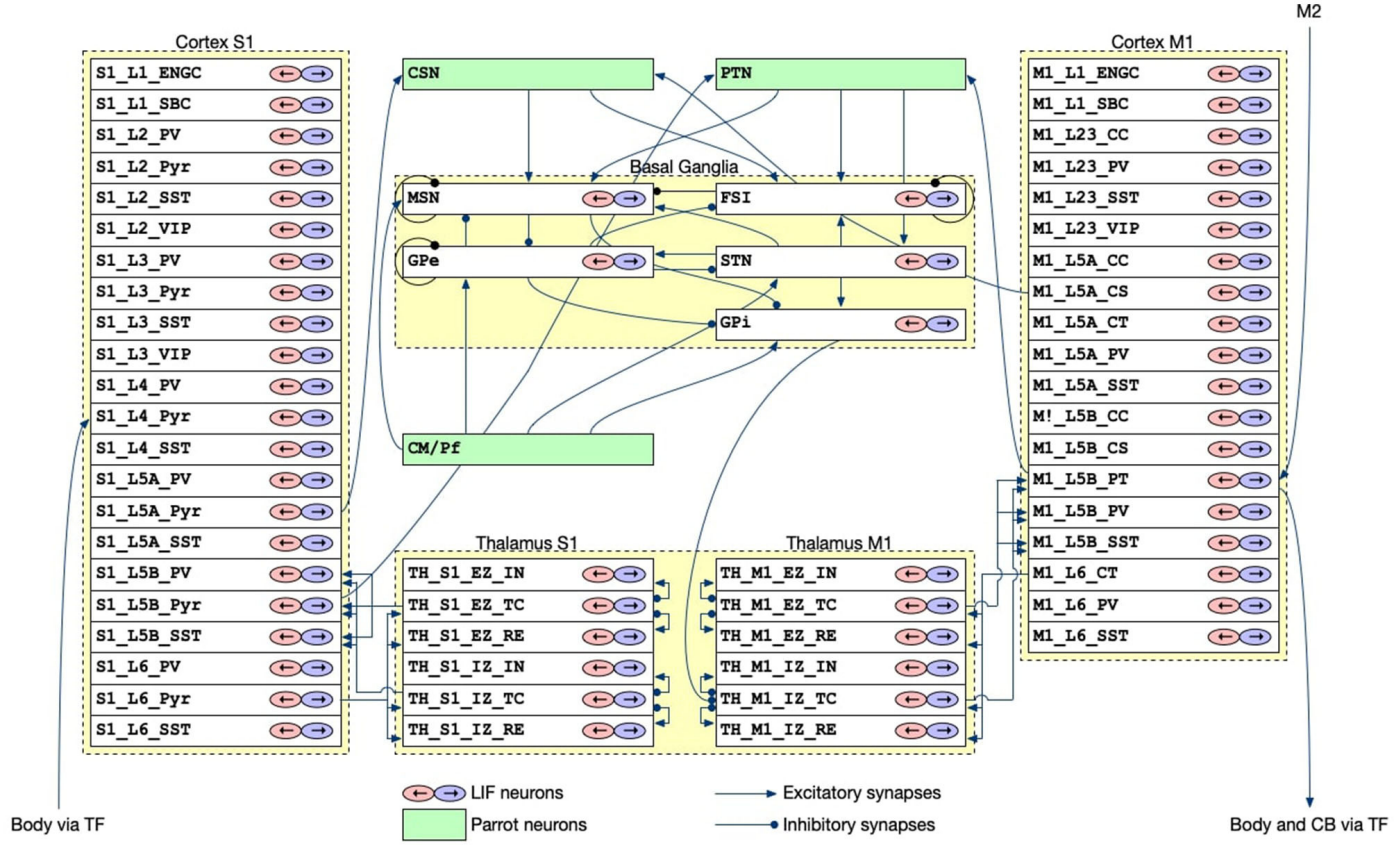
The architecture of the CBT model. The S1 and M1 models were composed of 22 and 19 layers for different cell types, respectively. Although there were a number of synaptic connections within S1 and M1, we omitted all such intra-regional connections for simplicity of the drawing. The other intra- and inter-layer connections are shown. Triangle and circle arrows represent excitatory and inhibitory connections, respectively. All white layers with two arrows contained LIF neurons, whereas Green layers are virtual “parrot” layers in the NEST simulator, where a “parrot" neuron simply lets spikes pass through from input neurons to output neurons.

The CBT model, which was developed previously (Gutierrez et al., 2020), is the main component of our brain-body-environment simulation (Figure 2). The model is also adopted by our previous study (Feldotto et al., 2022a) without any modification. Briefly, the model is a collection of various subregion models for M1, S1, BG, and TH, which were developed in previous computational works (Liénard and Girard, 2014; Igarashi et al., 2019; Girard et al., 2021). The parameters including axonal and synaptic delays, synaptic weights, time constants, and the numbers of neurons were set based on experimental data (Lev and White, 1997; Weiler et al., 2008). The cortical models have six layers in which various types of neurons exist (Table 1). The BG model consists of multiple nuclei such as the striatum containing medium spiny neurons (MSNs) and fast spiking interneurons (FSIs), external and internal capsules of globus pallidus (GPe and GPi), and subthalamic nucleus (STN; Table 1). The TH model is divided into excitatory and inhibitory subcortical input-dependent zones (EZ and IZ) containing various types of neurons (Table 1). The model was entirely implemented in the NEST simulator version 2.20.0 (Gewaltig and Diesmann, 2007) with 176,465 LIF neurons (conductance-based and current-based, depending on the regions), and the simulation was conducted using 12 cores in a single compute node of Fugaku. In this study, we used this model to generate motor commands to the body based on the desired goal movement from M2, while also receiving the feedback signals of the body states.

**Table 1:** Layers and neuron types in the CBT and CB models.

**Model**	**Layer**	**Neuron types**
	L1	ENGC, SBC
	L2	PV, Pyr, SST, VIP
	L3	PV, Pyr, SST, VIP
S1	L4	PV, Pyr, SST
	L5A	PV, Pyr, SST
	L5B	PV, Pyr, SST
	L6	PV, Pyr, SST
	L1	ENGC, SBC
	L2/3	CC, PV, SST, VIP
M1	L5A	CC, CS, CT, PV, SST
	L5B	CC, CS, PT, PV, SST
	L6	CT, PV, SST
BG		FSN, MSN, GPe, GPi, STN
TH	EZ	IN, TC, RE
	IZ	IN, TC, RE
CB		MF, CF, GrC, GoC, BC, SC, PKJ, DCN

ENGC, elongated neugliaform cell; SBC, single bouquet cell; PV neuron, parvalbumin-expressing neuron; Pyr neuron, pyramidal neuron; SST neuron, somatostatin-expressing neuron; VIP neuron, vasoactive intestinal peptide-expressing neuron; CC neuron, Corticocortical neuron; CS neuron, corticospinal neuron; CT neuron, corticothalamic neuron; PT neuron, pyramidal-tract neuron; FSI, fast-spiking interneuron; MSN, medium spiny neuron; GPe, external segment of globus pallidus; GPi, internal segment of globus pallidus; STN, subthalamic nucleus; EZ, excitatory subcortical input-dominant zone; IZ, inhibitory input-dominant zone; IN, interpositus nucleus; TC neuron, thalamocortical neuron; RE nucleu, thalamic reticular nucleus; MF, mossy fiber; CF, climbing fiber; GrC, granule cell; GoC, golgi cell; BC, basket cell; SC, stellate cell; PKJ, purkinje cell; DCN, deep cerebellar nucleus.

#### 2.2.3. CB model

The CB model, which was originally developed in our previous study (Kuriyama et al., 2021), was also included in the system to demonstrate (a) online adaptation capability of our brain-body-environment model and (b) flexibility of the system. The model was designed for real-time simulation of a cerebellar spiking network by harnessing GPUs, and has a capability of online gain adaptation for eye movement reflex called optokinetic response. A role of the CB model is to calculate a compensatory gain which amplifies motor commands generated by the CBT model. The details of the gain adaptation mechanisms are described in Section 2.6.

**Figure 3:**
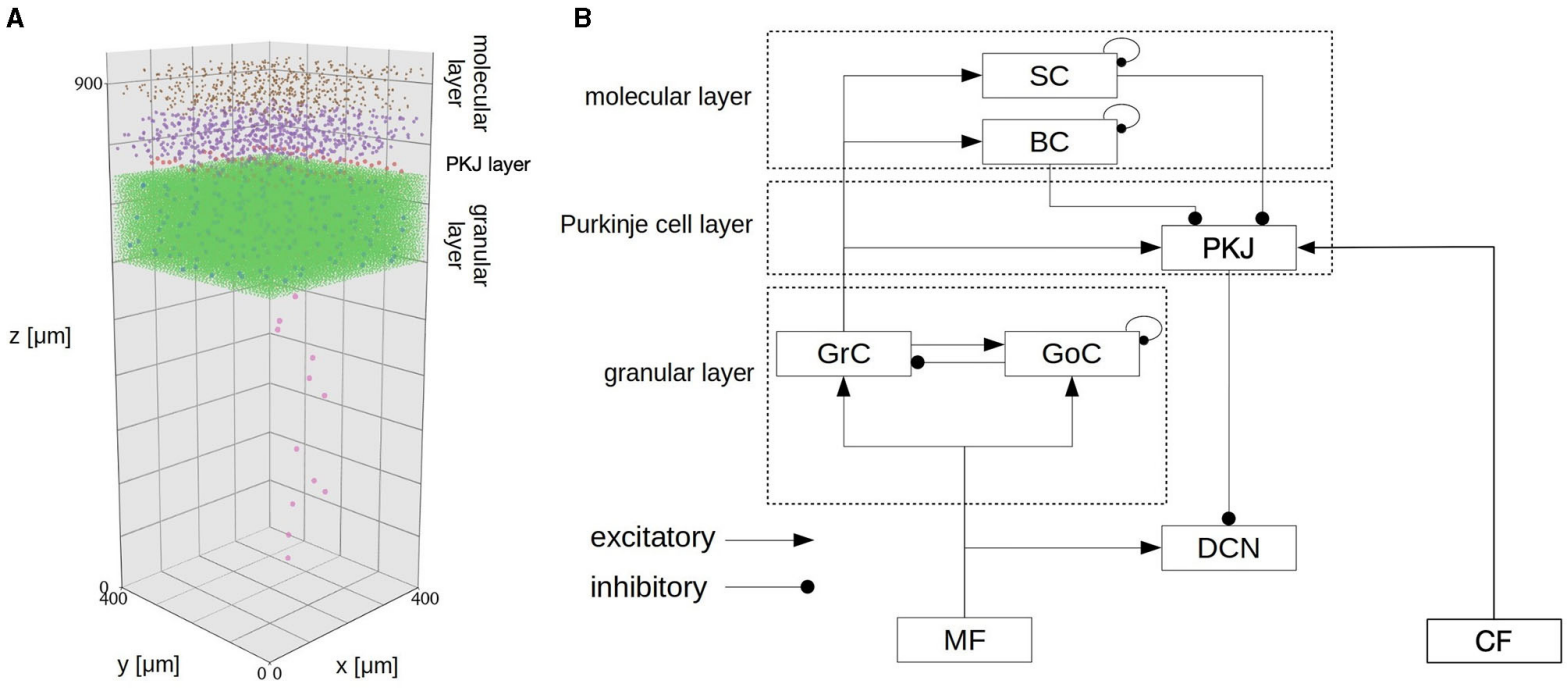
Spatial arrangement of neurons in the CB model. The figure was adapted from Fig. 1 of Kuriyama et al. (2021). **(A)** Cell placement in a 3D volume of 400 × 400 × 900 μ*m*^3^. Dots represent neurons except for CFs. Detailed explanation in our previous paper (Kuriyama et al., 2021). **(B)** Schematic of connectivity in the CB model. Abbreviations as listed in Table 1.

The model implements a 400 × 400 × 900μm^3^ volume of the cerebellar cortex and deep nuclei (Figure 3; Table 1) composed of 88,158 granule cells, 219 Golgi cells, 603 basket cells, 603 stellate cells, 69 Purkinje cells (PKJs), and 12 deep cerebellar nuclear neurons (DCNs), where all neurons were implemented as conductance-based LIF neurons. The model has two types of inputs: 7,073 mossy fibers (MFs) and 12 climbing fibers (CFs).

MFs connect to granule cells and DCNs, then granule cells extend parallel fibers (PFs) to Golgi cells, molecular layer interneurons (basket cells and stellate cells) and PKJs, and excite them. Golgi cells inhibit granule cells, whereas molecular layer interneurons inhibit PKJs. PKJs inhibit DCNs, which in turn provide the only outputs of the CB model. On the other hand, CFs connect and deliver instruction signals to PKJs. PKJs adapt their activity depending on the signals, and regulate DCN activity. MFs and CFs are implemented as Poisson spike generators with inhomogeneous firing rates. All parameters for neurons (e.g., membrane capacitance, leak conductance, threshold potential, etc.) and synapses (e.g., reversal potential, synaptic weight, and synaptic delay) were set the same as in the previous study (Kuriyama et al., 2021).

The CB model was implemented in C++ with CUDA (NVIDIA, 2023), and simulated on a local GPU cluster (an NVIDIA DGX Station with four NVIDIA V100 GPUs).

### 2.3. Body and environment model

**Figure 4:**
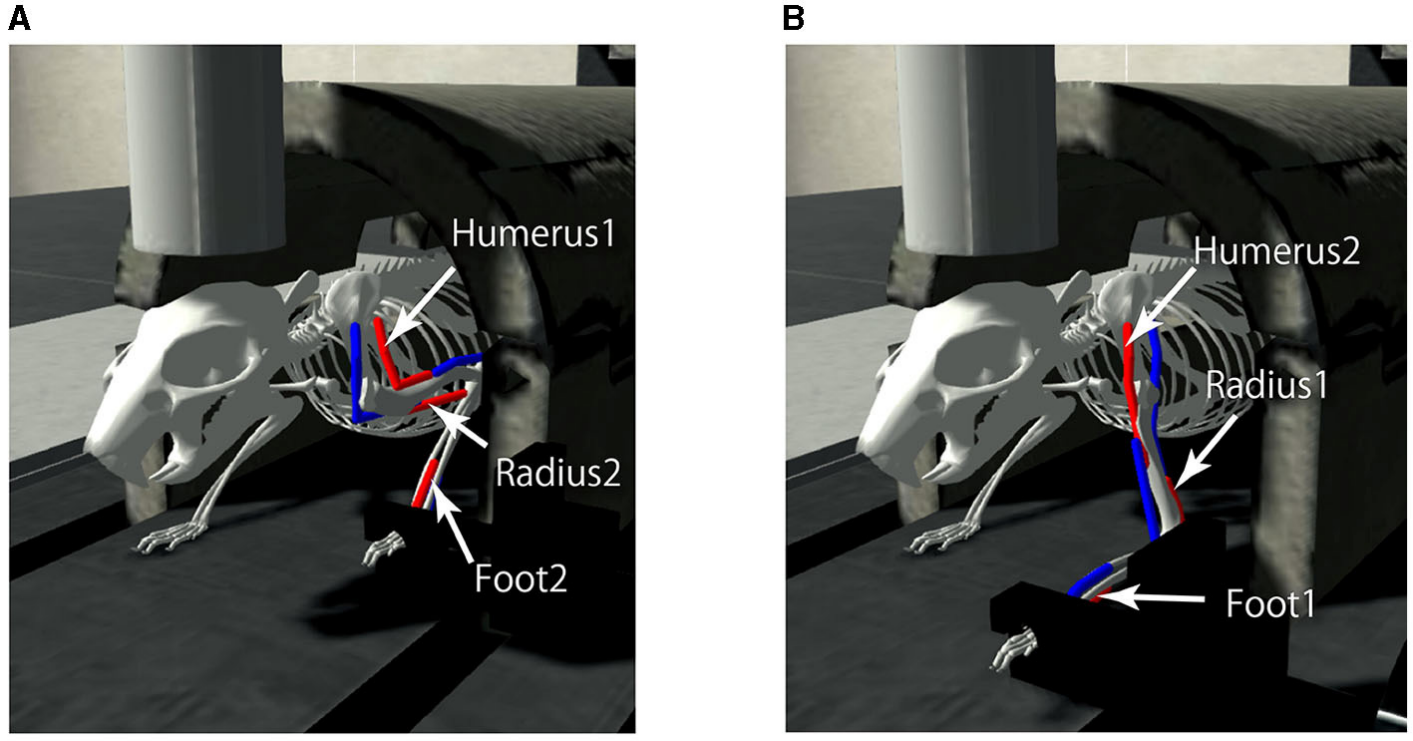
The mouse musculoskeletal model. The model has a movable forelimb composed of three joints and three pairs of antagonistic muscles (i.e., six muscles): Humerus 1 and 2, Radius 1 and 2, and Foot 1 and 2. The mouse pulls **(A)** or pushes **(B)** the lever attached to the forelimb, when the muscles contracted maximally, visualized in red.

A mouse musculoskeletal model was adapted from previous work (Allegra Mascaro et al., 2020) internally referred to as the “CDP1 Mouse Experiments" in the Neurorobotics Platform (Albanese et al., 2020) and used as the body-environment model. The model has a movable forelimb composed of three joints and three pairs of antagonistic muscles (i.e., six muscles): Foot1, Radius1, and Humerus 2 as extensors, and Foot2, Radius2, and Humerus1 as flexors. The mouse simulation model including the kinematic configuration and virtual muscles has been configured previously using the NRP Robot Designer (Feldotto et al., 2022b). Each muscle receives real numbers that represents the degree of activation. The contraction is calculated by the muscle dynamics ranging from 0 (completely relaxed) to 1 (maximum activation), which are the motor commands specified by the normalized population-averaged firing rates of L5B PT neurons in M1. Furthermore, a movable lever is attached to the forelimb. When the three extensors (Foot1, Radius 1, and Humerus2) are maximally contracted, the mouse pushes the lever maximally, whereas when the three flexors (Foot2, Radius 2, and Humerus1) are maximally contracted, the mouse pulls the lever maximally (Figure 4). The information of individual muscle lengths of the limb as muscle states are obtained and fed to L4 Pyr neurons in S1. The model simulation was conducted on a local GNU/Linux PC (Ubuntu 20.04.6 LTS; Intel(R) Xeon(R) CPU E5-2667 v4 @ 3.20GHz; 128 GB RAM).

### 2.4. Communication between the brain and body models

**Figure 5:**
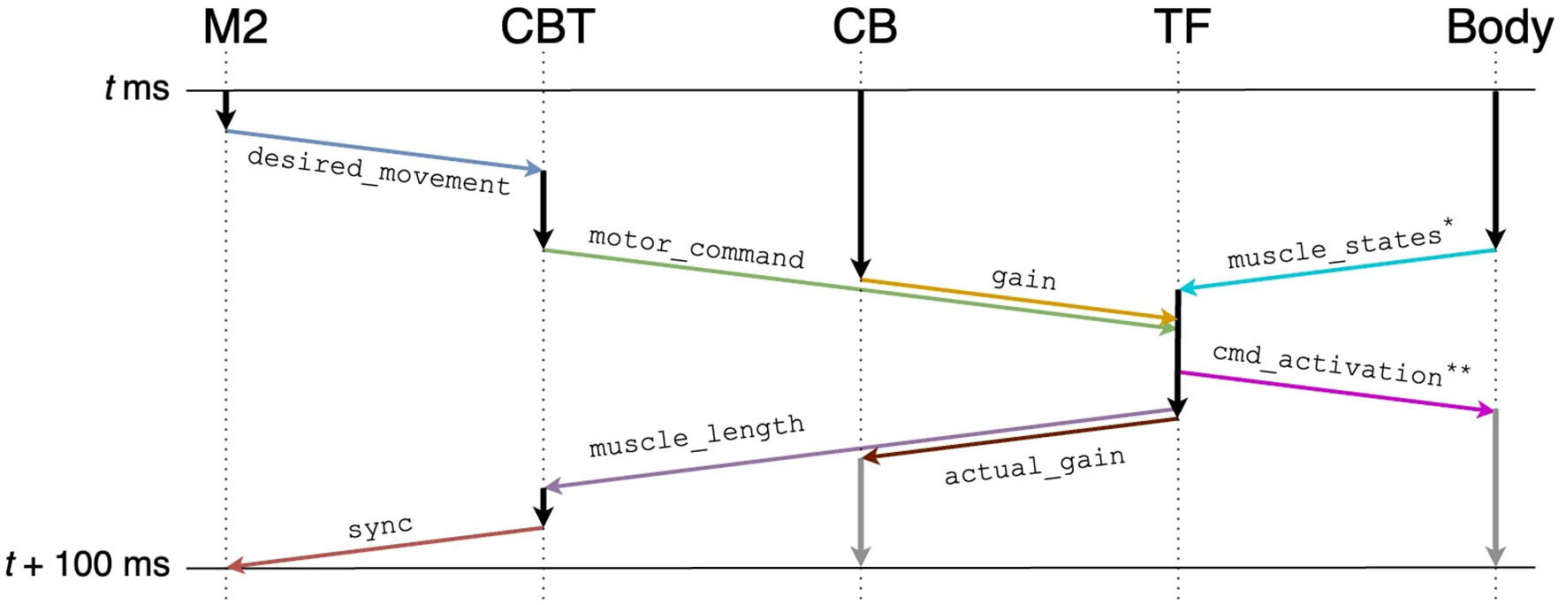
Communication diagram across components for each 100 ms of the physics simulation time. Arrows represent information flow across components, and in particular, colored arrows represent publication/subscription of ROS topics summarized in Table 2. Gray vertical arrows represent calculation after *t*+100 ms. Annotated topics (* and **) have full names (Table 2).

All models communicated with each other to achieve bidirectional simulation (Figures 1, 5). The source of the activation was the M2 model, which emitted two spike trains alternating periodically at about 0.5 Hz as desired movements. The spike trains were fed to L5B PT neurons in M1 (Figure 2), which in turn emitted similar spike trains that represent motor commands. The spike trains were propagated to the other layers of M1 and BG internally, and to CB and the body via TF, which could be regarded as a fictitious spinal cord. TF received normalized firing rates calculated by L5B PT neurons in M1, translated the firing rates into muscle activation levels within [0, 1], and fed the levels to the muscles. Then, muscles contracted and the mouse started to push and pull the lever repeatedly with the same period of the spiking activity of M2, namely, 2 s. The muscle length information was fed back to the L4 Pyr neurons in S1, which also exhibited similar alternating spike patterns. These mathematical forms can be found in the Supplementary material: how to convert firing rates of L5B PT neurons in M1 into muscle activation levels (Section 2) and how to convert muscle length into firing rates, which represent inputs to L4 Pyr neurons in S1 (Section 3). There are pathways from S1 to M1 including a pathway from L4 Pyr neurons in S1 → L5A Pyr neurons in S1 → CSN → MSN in BG → GPi in BG → M1 IZ TC neurons in TH → L5B PT neurons in M1 (Figure 2), thus closing the loop between the brain and the body. For simplicity in this study, inter-regional synaptic connections assumed certain time delays of milliseconds, but not several tens of milliseconds (Lemarechal et al., 2021), for spike transmission. Meanwhile, the normalized firing rates from L5B PT neurons in M1 were also fed to the mossy fibers in CB via TF. On the other hand, compensatory gain was calculated from the firing rates of the DCN and was sent to TF. The gain was adapted gradually during simulation for 10s.

To implement these communications across components, namely M2, CBT, CB, TF, and Body, we adopted ROS as the communication middleware suite for the present system.

### 2.5. Implementing the communication on ROS

To realize the message passing across components, we employed ROS which is a popular middleware suite used in the field of robotics (Quigley et al., 2009). ROS allows robots and controllers to “publish” or “subscribe” messages periodically via a queue called a “topic” for sharing the messages, through which they can communicate bidirectionally. For example, a controller publishes a message on desired movement direction to a topic, whereas a robot subscribes the message from the same topic and moves toward the direction. Similarly, the same robot can publish a message on the actual movement direction to the topic, whereas the controller subscribes the message from the same topic and compensate the direction. In ROS, a robot or a controller can be represented as a “ROS node.” Thus, ROS nodes communicate with each other while publishing/subscribing messages to topics. For the communication shown in Figure 5, we created topics summarized in Table 2. We defined the following topics: spike trains from the M2 model to the CBT model (desired_movement), the firing rate of M1 neurons to TF (motor_command), muscle activation from TF to muscles (cmd_activation), the current muscle length from the muscles to TF (muscle_states), the firing rates reflecting the antagonistic muscle lengths for Humerus1/2 to the CBT model (muscle_length), the compensatory gain from the CB model to TF (gain), the calculated actual gain from TF to the CB model (actual_gain), and a control signal from the CBT model to the M2 model to resume the simulation of M2 for the next 100 ms of the physics simulation time (sync). ROS messages were published every 1 s, and the network latency between the Fugaku and the local network was about 10 ms.

**Table 2:** Summary of ROS topics.

**Topic name**	**Type**	**Contents**
desired_movement	std_msgs/Float64MultiArray	M2 elapsed time, neuron IDs, spike times
motor_command	std_msgs/Float64MultiArray	CBT elapsed time, firing rates of L5B PT neurons in M1
gain	std_msgs/Float64MultiArray	CB elapsed time, gain
muscle_states ^*^	MuscleStates^***^	Current muscle lengths
actual_gain	std_msgs/Float64MultiArray	TF elapsed time, actual_gain, firing rates of L5B PT neurons in M1
muscle_length	std_msgs/Float64MultiArray	TF elapsed time, firing rates reflecting lengths for Humerus1, 2
cmd_activation ^**^	std_msgs/Float64	Muscle activation
sync	std_msgs/Int32	CBT elapsed time

^*^muscle_states has a full topic name /gazebo_muscle_interface/robot/muscle_states.

^**^ cmd_activation has a full topic nam /gazebo_muscle_interface/robot/name/cmd_activation for each name ∈ {Foot1, Foot2, Radius1, Radius2, Humerus1, Humerus2}.

^***^ MuscleStates has a full type name gazebo_ros_muscle_interface/MuscleStates.

To synchronize the brain model and the body-environment model, we elaborated our own TF. Specifically, all ROS topics sent to TF included information on the local elapsed time (Table 2). Then, TF detected the completion of the other components ecept M2 for each 100 ms of the physics simulation time. Finally, TF sent the information on the next target time (i.e., current time + 100 ms) back to them (Figure 5). For M2, the synchronization was not mediated by TF directly, but indirectly via the CBT model. Owing to the synchronization mechanism, simulation results were reproducible as long as the simulations were initialized with the same random seed.

The messages are passed over the Internet, and all ROS nodes must be in the same subnet by default. This means, ROS nodes in different subnets cannot communicate directly. This is a problem when a brain model and a body model run on different computers in different subnets as in our case. To address this issue, ROS provides a mechanism called rosbridge (ROS, 2022). Rosbridge is a communication protocol that allows a program without ROS functionality (rosbridge client) to connect to ROS nodes. Communication over rosbridge is made by wrapping ROS topics in JSON format, and then sending/receiving them to the rosbridge server using the WebSocket protocol. The rosbridge server, which is also a ROS node, relays communications over rosbridge. Because the WebSocket protocol is allowed to pass through firewalls in most organizations, rosbridge allows us to connect a brain model on Fugaku and a body-environment model on a local computer. Furthermore, libraries in Python and Javascript are available for rosbridge. We needed to use rosbridge for the M2 model and the CB model written in C++. For this purpose, we developed a C++ version of the rosbridge library called CppRosBridge (Omura, 2022). CppRosBridge is built to depend on external libraries as minimally as possible. In fact, it depends only on Jansson (Lehtinen, 2018) and websocketpp (Thorson, 2014) for JSON APIs and WebSocket communications, respectively.

### 2.6. Gain adaptation by the CB model

An essential role of the CB model for the entire system is to provide learning mechanisms based on simulated long-term depression (LTD) and potentiation (LTP) at parallel fiber-Purkinje cell synapses. The LTD at PF–PKJ synapses occurred by conjunctive activation of a CF and PFs (Sakurai, 1987). The mechanism decreases the synaptic efficacy, and so decreases PKJ activity. This decrease of PKJ activity leads to disinhibition of DCNs, so that the DCNs can increase their activation. The LTP at PF–PKJ synapses occurred by firing of a presynaptic PF only (Ito, 2001). This mechanism prevents synaptic efficacy from completely vanishing due to the LTD.

Specifically, the synaptic weights at PF–PKJ synapses were updated as folllows:


(5) $$\begin{array}{l} w_{{\text{PKJ}}_{i},{\text{PF}}_{j}}\left(t+\text{Δ}t\right)=w_{{\text{PKJ}}_{i},{\text{PF}}_{j}}\left(t\right)+\alpha {\text{PF}}_{j}\left(t\right)-\beta \overset{50}{\underset{s=0}{\sum }}{\text{CF}}_{i}\left(t\right){\text{PF}}_{j}\left(t-s\text{Δ}t\right), \end{array}$$

where *w*_PKJ_*i*_, PF_*j*__(*t*) is a synaptic weight between *i*th PKJ and *j*th PF, and PF_*j*_(*t*) (CF_*i*_(*t*)) take 1 if a PF of *j* th granule cell (a CF connects to *i* th PKJ) elicited a spike at time *t*, and 0 otherwise. The 2nd term on the RHS simulates LTP that occurred by firing of a presynaptic PF only. The 3rd term simulates LTD by conjunctive activation of a CF and a PF, that are active 0–50 ms earlier than the CF activation. Both α and β are learning coefficients for LTP and LTD, respectively. However, we only applied LTD in order to reduce the required simulation time for the CBT model. Consequently, parameters α and β were set to 0 and 0.4, respectively.

Equation (5) contains PF and CF spikes that need to be determined. First, PF spikes were emitted by granule cells in response to the MF spikes, which represent the efference copy of the motor command in M1. Specifically, the MF firing rate was calculated from normalized firing rates of L5B PT populations in M1 at the CBT model, and were received via TF. In our implementation, MFs were divided into two groups, and the firing rate of each group reflected that of one L5B PT population in M1 as follows:


(6) $$\begin{array}{l} \rho_{{\text{MF}}_{1}}=\{\begin{array}{ll} 4 & \text{if }\rho_{{\text{L5BPT}}_{\text{1}}}<\rho_{{\text{L5BPT}}_{\text{2}}},\\ 24 & \text{if }\rho_{{\text{L5BPT}}_{\text{2}}}<\rho_{{\text{L5BPT}}_{\text{1}}}. \end{array}\\ \rho_{{\text{MF}}_{2}}=\{\begin{array}{ll} 4 & \text{if }\rho_{{\text{L5BPT}}_{\text{2}}}<\rho_{{\text{L5BPT}}_{\text{1}}},\\ 24 & \text{if }\rho_{{\text{L5BPT}}_{\text{1}}}<\rho_{{\text{L5BPT}}_{\text{2}}}, \end{array} \end{array}$$

where *ρ*_MF_*x*__ is firing rate of MF group *x* ∈ {1, 2}, and *ρ*_L5BPT_*x*__ is firing rate of M1 L5B PT group *x* ∈ {1, 2}.

On the other hand, the firing rate of CFs, as error inputs, represented the difference between the desired and actual motor gain. Specifically, the firing rate was calculated by a monotonically increasing function of the difference between hard-corded desired gain and actual gain information received from TF. The firing rate was defined as follows:


(7) $$\begin{array}{l} \rho_{\text{CF}}=6\times \left(1-\tilde{G}\right), \end{array}$$

where *ρ*_CF_ is firing rate of CFs, $\tilde{G}\in \left[0,1\right]$ is a normalized actual gain calculated by TF. How to calculate the actual gain can be found in Section 4 of the Supplementary material.

The adaptive changes of PF–PKJ synaptic weights let DCNs to increase their firing rate, which is represented as compensatory gain *g*.


(8) $$g(t)=\{\begin{array}{ll} 1 & \text{if }\varphi_{\text{DCN}}(\text{t})\leq \text{0}.\text{85},\\ 1+10(\varphi_{\text{DCN}}(t)-0.85) & \text{if }\varphi_{\text{DCN}}(\text{t})>\text{0}.\text{85}. \end{array}\;$$

(9) $$\begin{array}{l} 10\frac{d\varphi_{\text{DCN}}\left(t\right)}{dt}=-\varphi_{\text{DCN}}\left(t\right)+\overset{N_{\text{DCN}}}{\underset{i}{\sum }}{\text{DCN}}_{i}\left(t\right), \end{array}$$

where *φ*_DCN_(*t*), *N*_DCN_, and DCN_*i*_(*t*) are temporal population activity of DCNs, the number of DCNs, and the *i*th DCN's spike activity which takes 1 if it elicited a spike at time *t*, and 0 otherwise, respectively.

### 2.7. The supercomputer Fugaku

For our distributed simulation, we used local computers as well as the supercomputer Fugaku (RIKEN, 2021), the Japanese flagship supercomputer, to run the M2 and CBT models. It consists of about 160,000 compute nodes, where each compute node comprises an ARM-based A64FX processor with 48 computational and four assistant cores, 32GB HBM2 memory, and a network port. All compute nodes are connected via a special interconnect Tofu6D. Each computational core of the A64FX processor can issue SIMD operations called Scalable Vector Extentions (SVEs) on 512-bit registers. Fugaku runs a GNU/Linux operating system based on Red Hat Enterprise Linux 8 for ARM. Spack packaging system is available to load necessary packages on demand. Most standard compilers and scripting languages including Python are installed. In fact, Python is a prioritized programming language on Fugaku for AI/ML purposes.

## 3. Results

### 3.1. Distributed bidirectional brain-body-environment simulation across multiple computers

**Figure 6:**
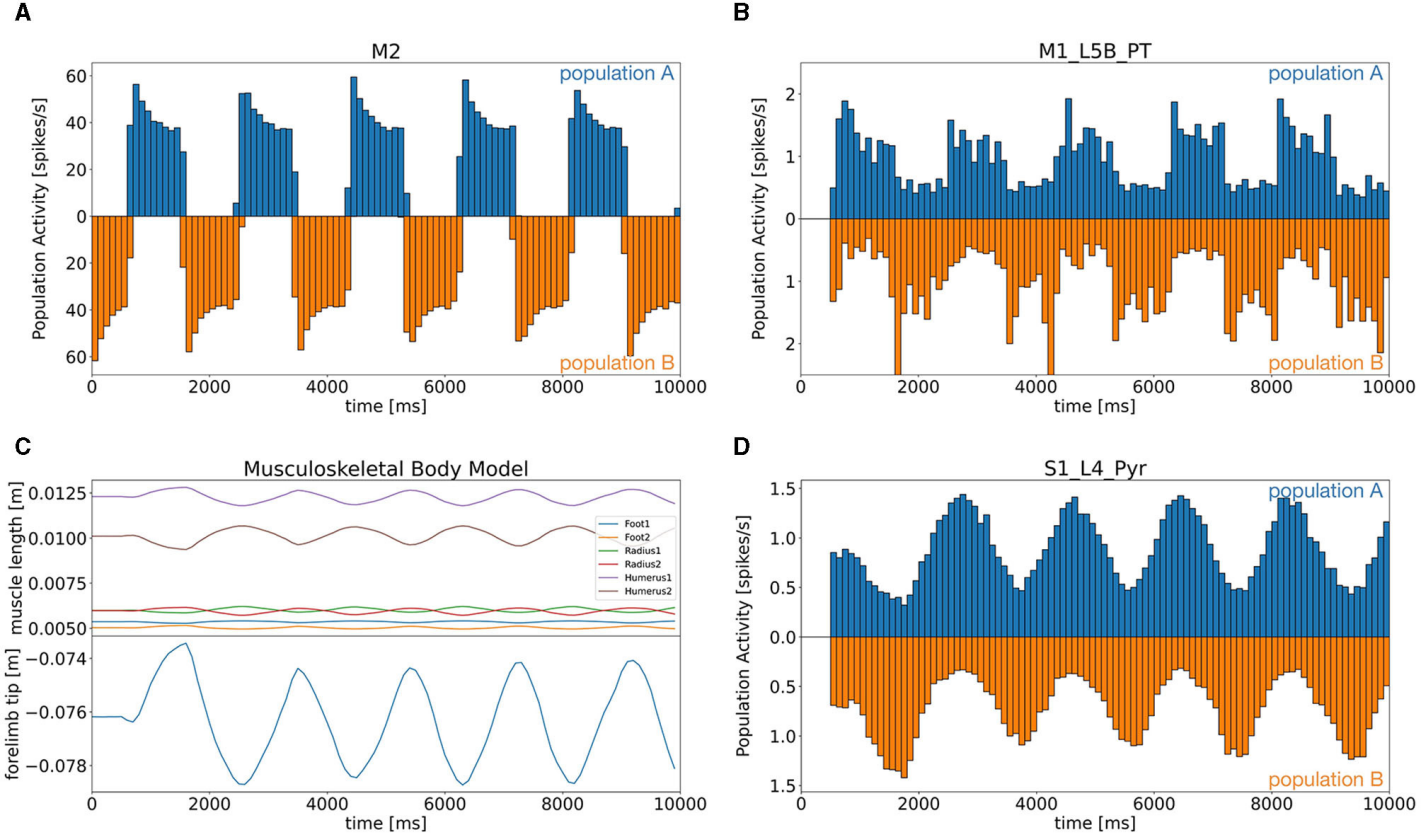
Population activity of the M2 model **(A)** and the CBT model [**(B)** L5B PT neurons in M1, **(D)** L4 Pyr neurons in S1], and plots of the body states **(C)**. **(A, B, D)** Firing rates for the two alternating populations were calculated and plotted separately above (population A) and below (population B) zero in a symmetric manner with different colors. **(C)** Top panel shows the lengths of all six muscles, whereas the bottom panel plots the lever position at the forelimb.

We first determined that all components were working coherently and synchronously to realize distributed bidirectional simulation between the brain and body models. In this simulation, we tested whether the mouse body model running on a local computer was able to push and pull a lever attached to the forelimb alternatively in response to the motor commands generated in the brain model running on Fugaku. Specifically, neurons in M2 were assumed to represent desired lever movements, which were alternating push/pull of the lever for every 1 s. Those neurons emitted spikes alternatively to represent the alternating lever movements (Figure 6A). Then, the spikes were fed to the downstream brain regions, and L5B PT neurons in M1 also emitted spikes alternatively with the same period to represent the motor commands for the desired lever movements (Figure 6B). The motor commands in M1 were sent via TF to the six antagonistic muscles, which lead the mouse body model to push/pull the lever alternatively with the same period (Figure 6C). Finally, L4 Pyr neurons in S1 received information on the body states, which were the muscle length, from the body model via TF, and also emitted alternating spike trains with the same period (Figure 6D). Thus, the communication between the brain and body models was not just one way but bidirectional. Raster plots of all neurons were shown in Supplementary Figure S1.

During the task, neurons emitted spikes alternatively or uniformly (Supplementary Figure S2). For example, the CB model received the firing rate of L5B PT neurons in M1 via TF as an efference copy of motor commands through MFs. All cerebellar neurons except DCNs showed alternating spike patterns that synchronized with M2 activity with a certain delay. Meanwhile, the spike activity of M1 neurons was propagated to neurons on different layers and in BG. Those neurons, however, emitted spikes rather uniformly: modulatory activities in time were subtle.

Overall, the brain and body models running on different computers worked coherently and synchronously, although those computers were located in different subnetworks. The synchronization was mediated by TF, whereas the communication across the subnetworks was realized by ROS and rosbridge.

### 3.2. Computational performance in the simulation

Because of the communication among ROS nodes across multiple computers distributed remotely, waiting time for synchronization in the communication may cause substantial problems. First, waiting time may slow down the simulations, because ROS nodes must wait for the update of topics. Moreover, too much waiting time may cause timeout at the system level and so simulations may be terminated accidentally. To investigate how much waiting time affect the entire simulation time, we measured the actual simulation runtime for all components.

**Figure 7:**
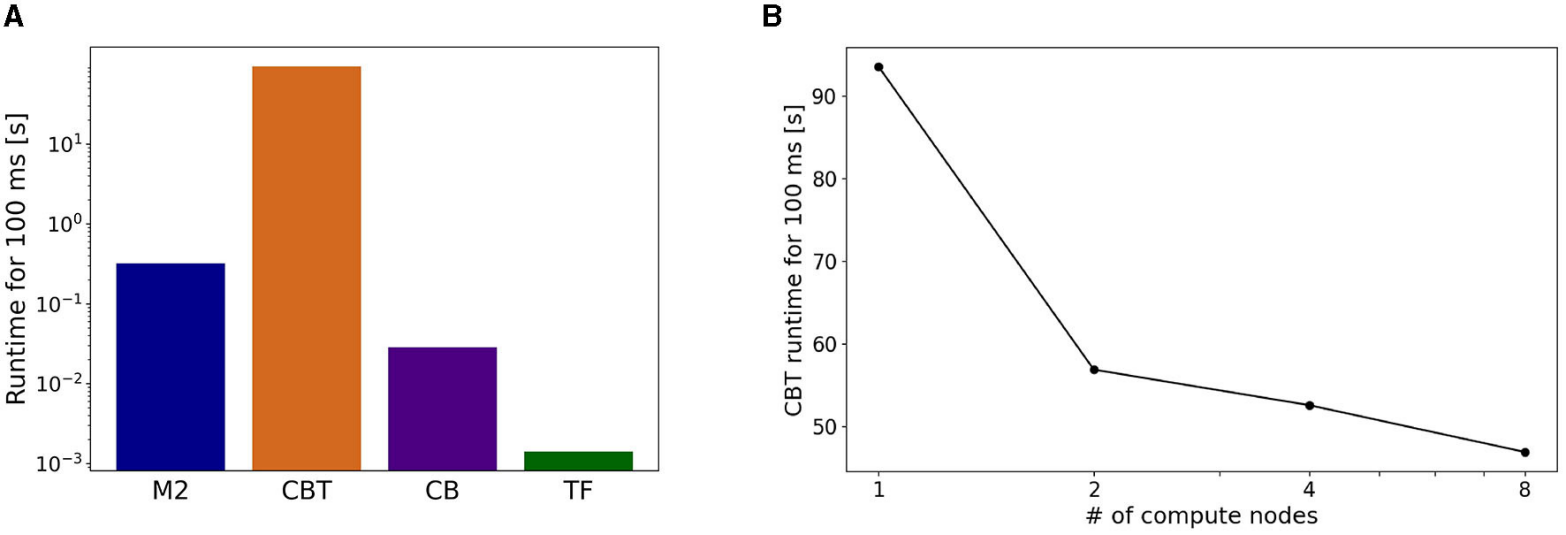
Breakdown of computational time. **(A)** Running time spent by each component in a simulation for 100 ms of the physics simulation time. **(B)** Strong scaling performance of the CBT model. Running time of the CBT model for the same 100 ms was measured while varying the number of compute nodes from 1 to 8.

On the actual runtime, the CBT model took the longest. The runtimes of M2 and CB models were less than a tenth of that of the CBT model, and that of TF was negligible (Figure 7A). Due to the imbalance of runtimes, M2, CB, and TF had to wait for the calculation of the CBT model. These results suggest that imbalance of runtime among all components affects the entire computational performance.

To address the imbalance of runtimes for the CBT model, we conducted parallel simulations for the CBT model by introducing more compute nodes. In other words, we measured the strong scaling property of the CBT model (Figure 7B). When the number of compute nodes was doubled from 1 to 2, we obtained almost 60% speed up. When the number of compute nodes was increased further, we still obtained slight speed up, but the scalability became worse.

### 3.3. Online gain adaptation by the CB model

**Figure 8:**
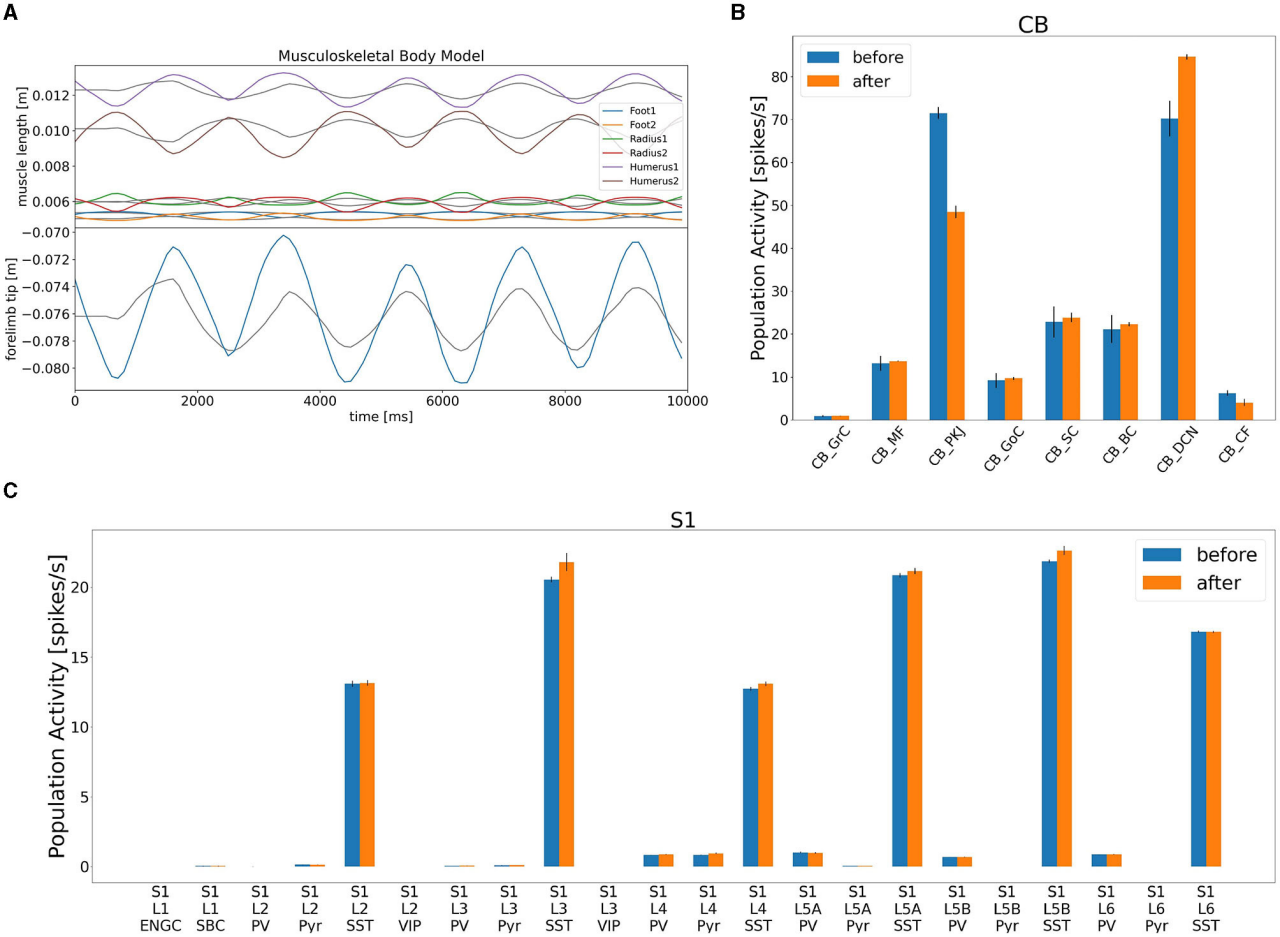
Gain adaptation during lever movements by the CB model. **(A)** Top panel shows the trajectory of lengths for all six muscles, whereas the bottom panel plots the trajectory of the lever position. **(B)** Change in population activity in the CB model. **(C)** Change in population activity in S1. Abbreviations listed as in the text.

After confirming such equilibrium network state, we turned on the learning mechanism in the CB model to adapt the movement gain of the lever push/pull (Figure 8). The initial gain was measured as 0.3, and we set the desired gain at 0.9, thereby making the movement three times larger. We spent 10 s for a learning period, and analyzed mean firing rates of CB neurons before and after learning (Figure 8A). We found that the mean firing rate of PKJs decreased from 71 to 49 Hz, on average, whereas that of DCNs increased from 71 to 84 Hz, on average (Figure 8B). The mean firing rate of the CFs also decreased from 6.8 to 3.4 Hz, on average, suggesting that the error between desired and actual gains decreased by learning. Finally, the increase of the DCN activity was translated to the compensatory gain, and made the lever movement about 2.3 times larger (Figure 8A). Furthermore, due to the gain increase, the activity of L4 Pyr neurons in S1 also increased (Figure 8C). These results suggest that our distributed simulation system allow us to study not just the entire network dynamics but also learning mechanisms that spanned across the network.

## 4. Discussion

In this study, we orchestrated various simulations of an M2 model written in C++ and a CBT model implemented in the NEST simulator on Fugaku, a CB model written in C++ and CUDA on a local GPU cluster, and a mouse musculoskeletal model in NRP on a local computer to achieve forelimb movements in a bidirectional manner by using ROS and rosbridge. The following two issues may summarize the novelty and usefulness of the present study.

First, owing to ROS, we were able to integrate multiple brain region models and a body-environment model running in multiple different simulators. ROS supports development in C++ and Python, and so both high-performance and easy-to-use neural simulators can be supported. Therefore other simulators such as NEURON (Carnevale and Hines, 2006) and Brian (Goodman and Brette, 2008) simulators will be included through their Python interfaces. Furthermore, our C++ version of the rosbridge library, which is newly developed by the present study, allows other high-performance neural simulators such as MONET (Igarashi et al., 2019) and GeNN (Yavuz et al., 2016) to be included as well. We will be able to further employ other body models such as a mouse full body model (Ramalingasetty et al., 2021), a human body model (Rajagopal et al., 2016), and even environment models such as an AI framework called Gymnasium (Farama Foundation, 2022). These will enable us to conduct simulation of more complex motor control tasks including reaching, grasping, and locomotion, suggesting the usefulness of our library.

Second, Fugaku is a powerful yet flexible and open supercomputer. We were able to compile and run the NEST simulator on Fugaku. Moreover, the CBT model on Fugaku was able to connect to other brain regions and body-environment models running on local computers over the Internet, demonstrating the openness of this supercomputer owing to rosbridge. Furthermore, our C++ version of the rosbridge library allowed the supercomputer Fugaku to participate in the distributed simulation system over the firewall, owing to the open architecture of the Fugaku system. In fact, this is the first demonstration of such distributed simulation that involves Fugaku. Furthermore, a human-scale spiking network model simulation on a MONET simulator has been reported, using all 160,000 compute nodes of Fugaku (RIKEN, 2021). Thus, simulation of a human brain-body model and an environment model will be possible from the aspect of compute resources required.

### 4.1. Related studies

As we demonstrated, ROS is a powerful technology to achieve such distributed simulations. However, various other choices exist.

To distribute the entire system, we chose ROS as the communication interface among various parallel and distributed computing frameworks. Message Passing Interface (MPI) is a standard framework that provides low-level Application Programming Interfaces (APIs) (Gropp et al., 1999). MPI allows us to run a single application program on multiple computers as if they act as a single computer with multiple processors. MPI also supports running multiple application programs simultaneously on multiple computers while passing messages with each other. Other frameworks shown below only support the latter usage. Remote Procedure Call (RPC) is a protocol used for client-server style communication, where a client submits a query to a server, and the server returns the answer to the client. A variant of RPC called gRPC has been developed by Google (gRPC, 2023), and used to build a large-scale application by combining a number of small applications called microservices.

In the field of computational neuroscience, MUlti SImulator Coordinator (MUSIC) is a standard choice to connect various neural simulators such as NEST and NEURON (Djurfeldt et al., 2010). MUSIC itself is implemented in MPI. There is an attempt to enhance interoperability between MUSIC and ROS (Weidel et al., 2016). In the field of robotics, OpenRTM-aist is a promising choice besides ROS, which aims to let multiple components communicate on a robot. OpenRTM-aist is built on an RPC-based architecture called Common Object Request Broker Architecture (CORBA) (Pope, 1998). These are also called robot middleware occasionally. ROS has been also used in the interdisciplinary area between neuroscience and robotics such as a brain-machine interface (Tonin et al., 2022).

To develop and maintain distributed brain-body-environment simulation, the framework must be (1) available, where implementations in various programming languages are provided, (2) maintained, where the development is active, (3) usable, where the framework can be adopted to our applications easily, and (4) reachable, where the communication can pass through firewalls. Notably, (4) could be problematic for the previously mentioned frameworks, because they were actually assumed to be used within the same subnetwork. The combination of ROS and rosbridge suffices all the four issues by using WebSocket.

Message Queuing Telemetry Transport (MQTT) is another protocol developed for IoT devices (MQTT.org, 2022). MQTT supports a publisher/subscriber-style message passing, which is similar to ROS. MQTT also supports WebSocket to go over firewalls (MQTT over WebSocket). Further investigation will be necessary to adopt MQTT rather than ROS.

### 4.2. Limitations

Although we successfully demonstrated the distributed simulation, several limitations and shortcomings exist. First, the most serious problem is the existence of TF as a single point of failure. All communications between the brain and body models must pass through TF. While TF can synchronize all the models, this suggests that our simulations were distributed, but still centralized at TF. Removing TF is a technical challenge to achieve a truly decentralized system. One possible way is to adopt peer-to-peer communication protocols. Second, controlling the entire system is still complicated. In particular, debugging a distributed system like the present system is quite difficult for standard neuroscientists like us. We need a sophisticated debugging system for such distributed systems.

### 4.3. Perspectives

Nevertheless, we foresee that distributed simulations are inevitable. As the size and complexity of models increase, there will be no way to simulate the entire system on a single computer. We must move on to distributed simulation at some point. Moreover, distributed simulation will also be necessary and helpful to include more scientists in developing an integrated and large-scale model like the present model.

For simplicity, the brain model developed in this study does not consider the inter-regional time delays reflecting white matter propagation. This problem can be addressed by adjusting the cycle of exchanging ROS messages between brain regions and adding the time delays reflecting the results of recent works (e.g., Lemarechal et al., 2021) to the spike times. As a result, we may investigate a role of time delays in synaptic transmission for shaping emergence of brain dynamics such as brain-wide oscillation and synchronization (Fries, 2005). Moreover, we will also be able to even incorporate more realistic ion channels and spatial structure, connectomics, glia, and so on. These will be the basis for a digital twin of living systems.

## 5. Conclusion

We developed a distributed simulation system for a spiking network model of the cortico-basal ganglia-cerebellar-thalamic circuit and a mouse musculoskeletal model on multiple computers including Fugaku over the Internet. Computer simulation of such a large-scale embodied model will provide the means to virtualize neurosciences and provide digital twins of living systems.

## Data availability statement

The original contributions presented in the study are included in the article/Supplementary material, further inquiries can be directed to the corresponding author.

## Author contributions

YK: Writing—original draft, Formal analysis, Investigation, Methodology, Visualization. RK: Writing—original draft, Formal analysis, Investigation, Methodology, Visualization. SO: Writing—original draft, Methodology, Software. CG: Writing—review and editing, Software. ZS: Writing—review and editing, Software. BF: Writing—review and editing, Funding acquisition. UA: Writing—review and editing, Funding acquisition, Software. AK: Writing—review and editing, Funding acquisition, Software. TY: Writing—review and editing, Investigation. TH: Writing—review and editing, Investigation. FM: Writing—review and editing, Funding acquisition, Software. JI: Writing—review and editing, Software. KD: Writing—review and editing, Software. TY: Conceptualization, Funding acquisition, Supervision, Writing—original draft.
