# Supplementary material for: Embodied bidirectional simulation of a spiking cortico-basal ganglia-cerebellar-thalamic brain model and a mouse musculoskeletal body model distributed across computers including the supercomputer Fugaku
Source: Front Neurorobot. 2023 Oct 5;17:1269848. doi: 10.3389/fnbot.2023.1269848 (PMC10585105; doi:10.3389/fnbot.2023.1269848)
Supplement: Supplementary file 2 [file Data_Sheet_1.PDF]

# Supplementary Material

## 1 MODIFICATION OF THE CDP1 MOUSE EXPERIMENT IN NRP

To use the mouse body model provided on NRP in a simplistic way, we modified the simulation code of the CDP1 mouse experiment (Allegra Mascaro et al., 2020) as follows. First, in order to stop the original state machine which controls a sled behavior, we modified `MouseCDP1Control.exd`. The `MouseCDP1Control.exd` written in Python defines a state machine for an experimental control, and also controls a sled of the lever. We modified an `execute()` method of `SledMoveForwardFixedSpeed` class to make the state machine stay permanently in a specific state. For the state, we chose `SledMoveForwardFixedSpeed` state, because the state machine reaches that state after initializing the state machine. Specifically, we commented out `sled_control.command_sled_position(pos)` line and also commented out an if-else statement in a while loop on the method, so that the state never transitions and the sled will be affected only by the body movement. We should note that PID parameters of the sled were set to (40, 10, 10). The state machine runs on an independent thread so that the infinite loop has no effect on the our experiment. Second, we wrote our original `spinalcordtf.py`. Third, we added a timestep tag in a position of the sibling of the bodyModel tag in `bibi_configuration.bibi` to set the communication time step to 100 ms.

## 2 HOW TO CONVERT FIRING RATE AND GAIN INTO CMD ACTIVATION

The topic `cmd_activation`, which represents the degree of muscle activation, was calculated at TF from the firing rates of two groups of L5B PT neurons in M1 and the gain information provided by CB. First, the difference between the firing rates of the two neuron groups in M1 was calculated as follows:

$$d(t) = \rho_{\mathrm{L5BPT}_1}(t) - \rho_{\mathrm{L5BPT}_2}(t), \tag{S1}$$

where $\rho_{\mathrm{L5BPT}_1}(t), \rho_{\mathrm{L5BPT}_2}(t)$ are firing rates of two populations of L5B PT neurons in M1, $d(t)$ is the difference. Then, the `cmd_activation` $s(t)$ was calculated for each $\Delta t$ as follows:

$$s(t + \Delta t) = g(t) \times (s(t) + d(t)/z), \tag{S2}$$

where $g(t)$ is the compensatory gain provided by CB, $z$ is a normalized constant set at 10, and $d(t)$ is calculated by Eq. (S1). If $s(t) \geq 0$, then the values of $s(t)$ and 0 were sent to the three extensors and flexors of the mouse body model, respectively. If $s(t) < 0$, then the values of $-s(t)$ and 0 were sent to the three flexors and extensors, respectively.

## 3 HOW TO CONVERT MUSCLE LENGTH INTO FIRING RATE

Real animals have organs of proprioception that can detect muscle length in their muscles called muscle spindles. Muscle spindles are wrapped around nerve fibers called Ia afferents. Blum et al. (2017) proposed a method to convert muscle lengths into firing rates of Ia afferents, but for simplicity in this study, the following procedure was used to convert muscle lengths to firing rates in TF.

We chose Humerus1 and 2, which are a flexor and an extensor for a shoulder. Lagrange interpolation was used so that the firing rate was set at 100 Hz when the muscles were extended maximally, at 0 Hz when the

muscles were contracted maximally, and at 50 Hz when the muscles were exactly in the mid-length. In other words, Humerus1 provided the highest firing rate when the lever was fully pushed, and Humerus2 the highest firing rate when the lever was fully pulled. Therefore, the equation for translating muscle length into firing rates is represented as follows:

$$\begin{aligned} \mathrm{fr1}(t) = \Bigg| & 50 \times \frac{(\mathrm{Humerus1}(t) - \mathrm{fwd1})(\mathrm{Humerus1}(t) - \mathrm{bwd1})}{(\mathrm{center1} - \mathrm{fwd1})(\mathrm{center1} - \mathrm{bwd1})} \\ & + 100 \times \frac{(\mathrm{Humerus1}(t) - \mathrm{fwd1})(\mathrm{Humerus1}(t) - \mathrm{center1})}{(\mathrm{bwd1} - \mathrm{fwd1})(\mathrm{bwd1} - \mathrm{center1})} \Bigg|, \\ \mathrm{fr2}(t) = \Bigg| & 100 \times \frac{(\mathrm{Humerus2}(t) - \mathrm{center2})(\mathrm{Humerus2}(t) - \mathrm{bwd2})}{(\mathrm{fwd2} - \mathrm{center2})(\mathrm{fwd2} - \mathrm{bwd2})} \\ & + 50 \times \frac{(\mathrm{Humerus2}(t) - \mathrm{fwd2})(\mathrm{Humerus2}(t) - \mathrm{bwd2})}{(\mathrm{center2} - \mathrm{fwd2})(\mathrm{center2} - \mathrm{bwd2})} \Bigg|, \end{aligned} \tag{S3}$$

where Humerus$\{1, 2\}$(t) is the current muscle length of Humerus$\{1, 2\}$, whereas fwd$\{1, 2\}$, bwd$\{1, 2\}$, and center$\{1, 2\}$ are constants that represent the lengths when the lever is maximally pushed, pulled, and located at the center, respectively. The actual parameter values are fwd1 = 0.01085, bwd1 = 0.0134, center1 = 0.01235, fwd2 = 0.0115, bwd2 = 0.00825, and center2 = 0.010.

The two firing rates calculated by the lengths of Humerus1 and Humerus2 were sent to the CBT model via the topic `muscle_length`. After that, based on the firing rates, two groups of 1,000 Poisson spikes were generated, and were fed to S1 L4 Pyr neurons through random connections, where the in-degree for each neuron was set to 5.

## 4 HOW TO CALCULATE ACTUAL GAIN

First, we defined the distance $D(t)$ between maximum and minimum values of the foot position in the last push/pull cycle divided by a hard-corded initial distance:

$$D(t) = \frac{\max_u (\mathrm{Foot}(u)) - \min_u (\mathrm{Foot}(u))}{\text{initial distance}}, \quad u \in [t_1, t_2], \tag{S4}$$

where $\mathrm{Foot}(t)$ is the foot position, $t_1$ and $t_2$ are start and end time of the cycle, respectively. The value of $D(t)$ falls within the range $[1, 3]$. Then, the actual gain $\widetilde{G}(t)$ was set as the normalized value of $D(t)$ as follows:

$$\widetilde{G}(t) = \frac{D(t) - 1}{2}. \tag{S5}$$

### 4.1 Figures

### 4.2 Movies

A movie showing that the mouse pushes and pulls the lever alternatively is available (Mov S1).

## REFERENCES

Allegra Mascaro, A. L., Falotico, E., Petkoski, S., Pasquini, M., Vannucci, L., Tort-Colet, N., et al. (2020). Experimental and computational study on motor control and recovery after stroke: Toward a constructive

loop between experimental and virtual embodied neuroscience. *Frontiers in Systems Neuroscience* 14. doi:10.3389/fnsys.2020.00031

Blum, K. P., Lamotte D'Incamps, B., Zytnicki, D., and Ting, L. H. (2017). Force encoding in muscle spindles during stretch of passive muscle. *PLOS Computational Biology* 13, 1–24. doi:10.1371/journal.pcbi.1005767

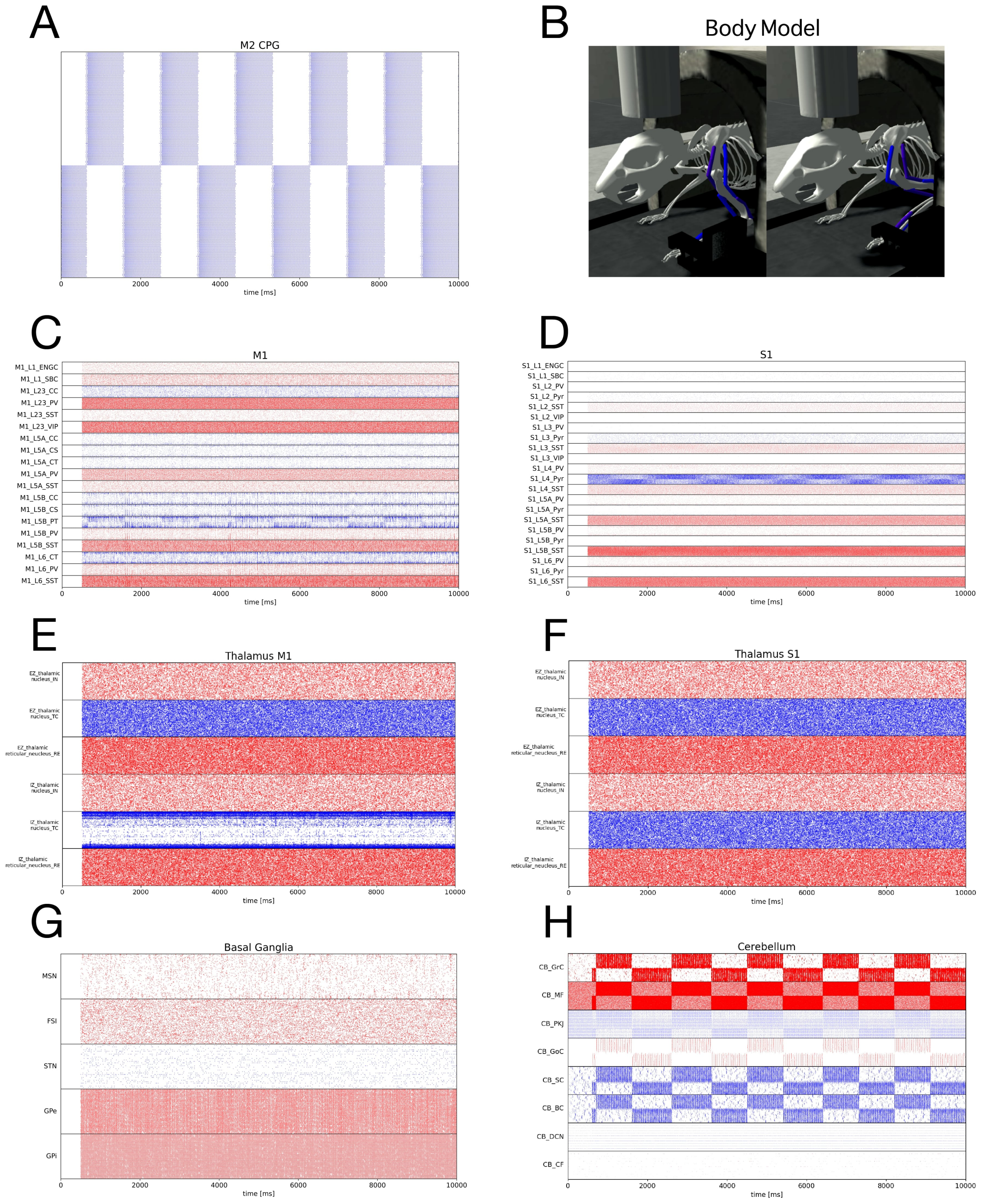


**Figure S1.** System dynamics. A and C-F: Spike rasters of all neuron types, A: M2; C: M1; D: S1; E: TH_M1; F: TH_S1; G: BG; H: CB. Simulations for 10,000 ms ( = 10 s) were performed to issue alternating lever movements 5 times. In the CBT model, spikes during the first 500 ms were omitted to discard the transient dynamics. B: forelimb movement: The left and right panels represent two arm positions when a mouse maximally pushes or pulls the lever, respectively.

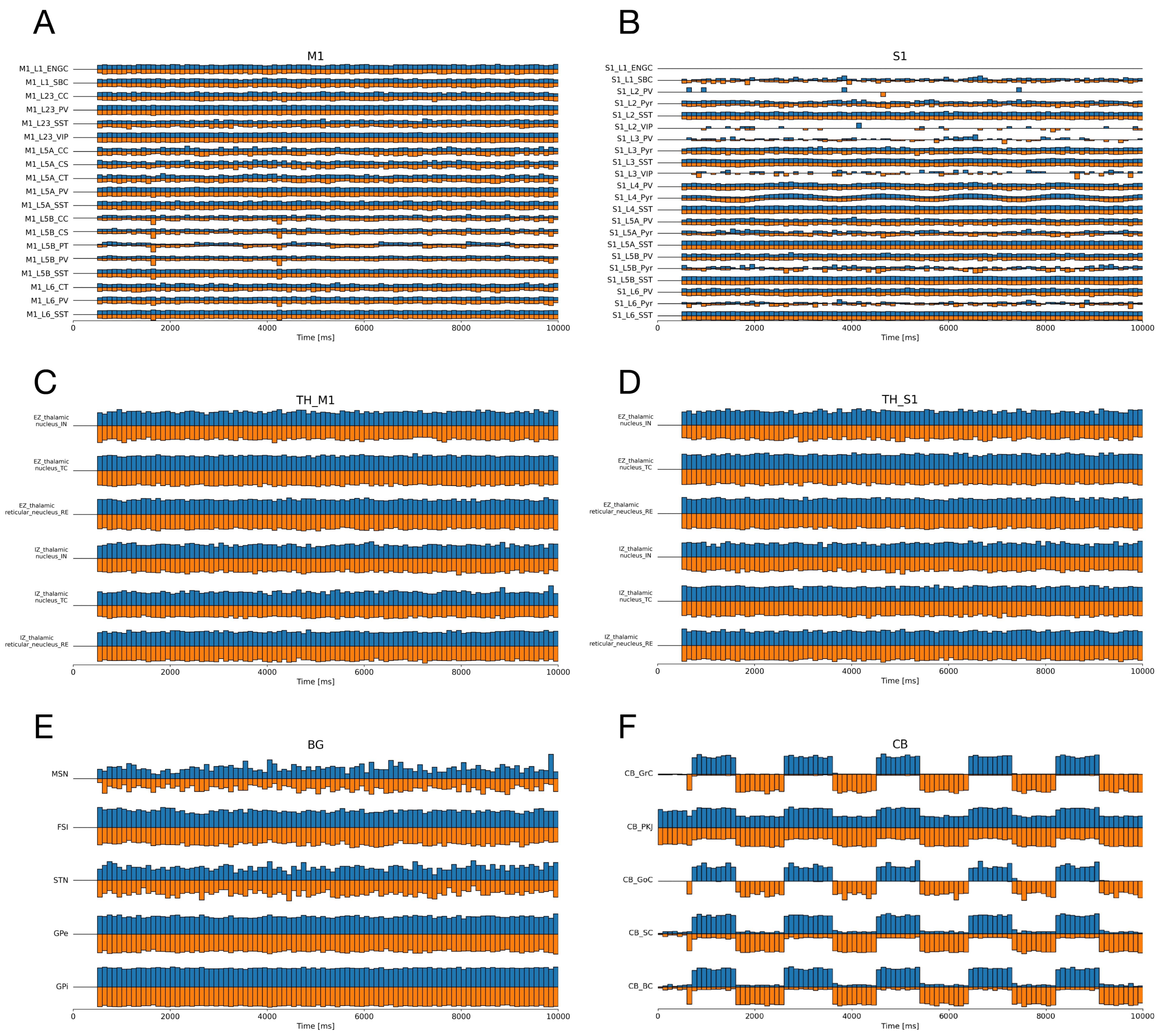


**Figure S2.** Population activity of the CBT model (A: M1; B: S1; C: TH_M1; D: TH_S1; E: BG), and that of the CB model (F). Firing rates for the two alternating populations were calculated and plotted separately with different colors.
