# Supplementary figures and images for: Embodied bidirectional simulation of a spiking cortico-basal ganglia-cerebellar-thalamic brain model and a mouse musculoskeletal body model distributed across computers including the supercomputer Fugaku
Source: Front Neurorobot. 2023 Oct 5;17:1269848. doi: 10.3389/fnbot.2023.1269848 (PMC10585105; doi:10.3389/fnbot.2023.1269848)

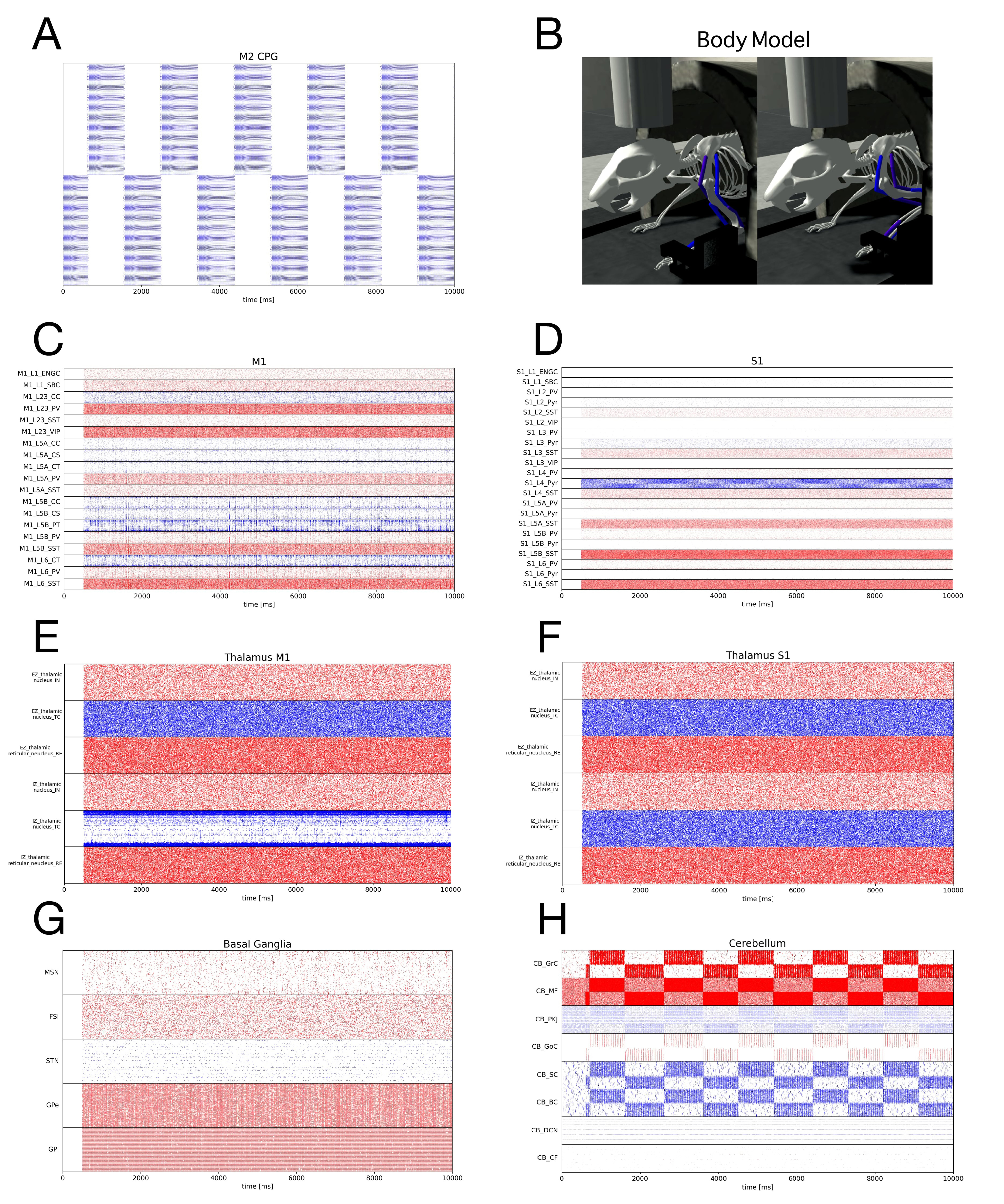

Supplement: Supplementary file 3 [file Image_1.JPEG]

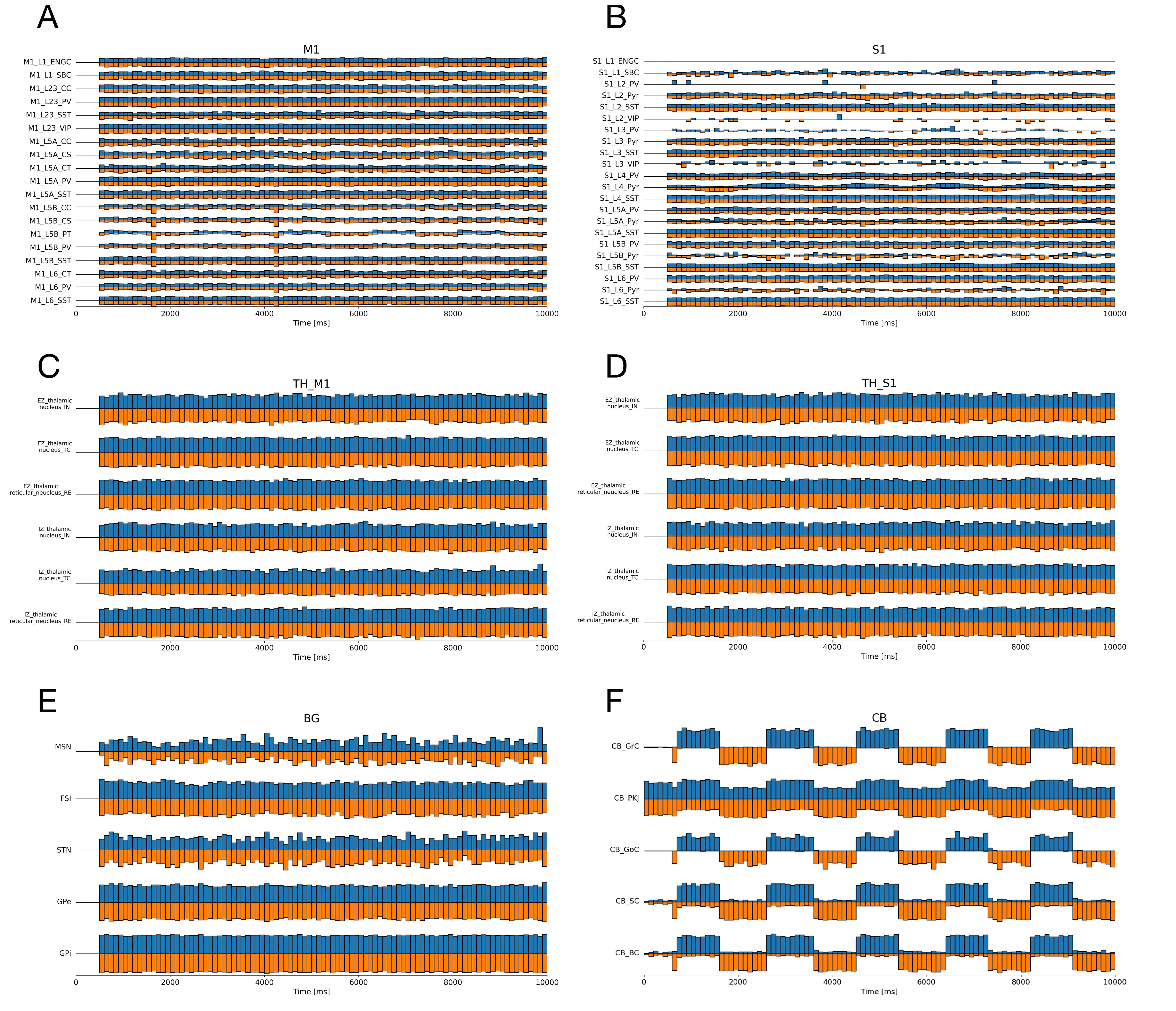

Supplement: Supplementary file 4 [file Image_2.JPEG]
